# Supplementary material for: Reasons Behind (Un)Healthy Eating Among School-Age Children in Southern Peru
Source: Foods. 2025 Jan 21;14(3):348. doi: 10.3390/foods14030348 (PMC11817301; doi:10.3390/foods14030348)
Supplement: Supplementary file 1 [file foods-14-00348-s001.zip › foods-3341730-supplementary.pdf]

Table S1. Calculation of the CSI of healthy foods mentioned by parents (own elaboration).

Food	Sum of Ranks	Frequency	Average Position (Eq.1)	Cognitive Salience Index (Eq.2)
Fish	52	23	2.26	0.46
Stews	26	13	2.00	0.29
Quinoa	26	13	2.00	0.29
Fruit	36	15	2.4	0.28
Vegetable	33	14	2.36	0.27
Milk	45	16	2.81	0.25
Lentils	20	9	2.22	0.18
Carrot	27	10	2.7	0.16
Broccoli	10	6	1.66	0.16
Liver	17	7	2.43	0.13
Meat	29	9	3.22	0.13
Orange	13	6	2.16	0.12
Banana	14	6	2.33	0.11
Spinach	15	6	2.5	0.10
Avocado	16	6	2.66	0.10
Salad	22	7	3.14	0.10
Cereal	17	6	2.83	0.09
Organ meat	9	4	2.25	0.08
Egg	17	5	3.4	0.06
Oats	11	4	2.75	0.06
Potatoes	11	4	2.75	0.06
Wheat	12	4	3	0.06

Prices were collected by the authors from December 22 to 24, 2024, between 11 AM and 1 PM, in local markets located in Moquegua (Mercado Central de Moquegua and Mercado Carillo) and Juliaca (Mercado Santa Bárbara and Mercado Túpac Amaru). To ensure representativeness, three stalls were consulted in each market for every product. The average of the reported prices was then calculated, reflecting typical costs for consumers in these regions. Table 1 displays the average prices of healthy foods in the markets of Moquegua and Juliaca, while Table 2 shows the average prices of unhealthy foods in the same areas.

Table S2. Average Prices of Healthy Foods ($/kg) in Markets of Moquegua and Juliaca (own elaboration).

Healthy food	Moquegua Price ($/kg)		Juliaca Price ($/Kg)	
	**Mercado Central de Moquegua**	**Mercado Carillo**	**Mercado Santa Bárbara**	**Mercado Túpac Amaru**
Fish (Jurel)	2.28	2.14	3.70	4.02
Stews (Beans, fava beans)	2.41	2.28	1.87	1.74
Quinoa	3.75	4.02	2.14	2.41
Liver	4.82	4.55	2.68	2.94
Lettuce	0.40	0.40	0.23	0.21
Olluco	1.34	1.20	0.93	0.93
Oca	1.1	1.1	0.80	0.80
Apple	1.07	1.07	0.94	1.07
Orange	0.67	0.53	0.53	0.53
Banana	1.34	1.34	1.20	1.34

Table S3. Average Prices of Packaged Unhealthy Foods ($/piece) in Markets of Moquegua and Juliaca (own elaboration).

Product (Per unit)	Moquegua Price ($/per piece)		Juliaca Price ($/per piece)	
	**Mercado Central de Moquegua**	**Mercado Carillo**	**Mercado Santa Bárbara**	**Mercado Túpac Amaru**
Chocolate	0.26	0.26	0.26	0.26
Candies	0.08	0.08	0.08	0.08
Bread	0.13	0.13	0.10	0.10
